# Speech Development Between 30 and 119 Months in Typical Children I: Intelligibility Growth Curves for Single-Word and Multiword Productions

**DOI:** 10.1044/2021_JSLHR-21-00142

**Published:** 2021-09-07

**Authors:** Katherine C. Hustad, Tristan J. Mahr, Phoebe Natzke, Paul J. Rathouz

**Affiliations:** a Department of Communication Sciences and Disorders, University of Wisconsin–Madison; b Waisman Center, University of Wisconsin–Madison; c Department of Population Health, Dell Medical School, The University of Texas at Austin

## Abstract

**Purpose:**

We extended our earlier study on normative growth curves for intelligibility development in typical children from 30 to 119 months of age. We also determined quantile-specific age of steepest growth and growth rates. A key goal was to establish age-specific benchmarks for single-word and multiword intelligibility.

**Method:**

This cross-sectional study involved collection of in-person speech samples from 538 typically developing children (282 girls and 256 boys) who passed speech, language, and hearing screening measures. One thousand seventy-six normal-hearing naïve adult listeners (280 men and 796 women) orthographically transcribed children's speech. Speech intelligibility was measured as the percentage of words transcribed correctly by naive adults, with single-word and multiword intelligibility outcomes modeled separately.

**Results:**

The age range for 50% single-word intelligibility was 31–47 months (50th–5th percentiles), the age range for 75% single-word intelligibility was 49–87 months, and the age range for 90% intelligibility for single words was 83–120+ months. The same milestones were attained for multiword intelligibility at 34–46, 46–61, and 62–87 months, respectively. The age of steepest growth for the 50th percentile was 30–31 months for both single-word and multiword intelligibility and was later for children in lower percentiles. The maximum growth rate was 1.7 intelligibility percentage points per month for single words and 2.5 intelligibility percentage points per month for multiword intelligibility.

**Conclusions:**

There was considerable variability in intelligibility development among typical children. For children in median and lower percentiles, intelligibility growth continues through 9 years. Children should be at least 50% intelligible by 48 months.

**Supplemental Material:**

https://doi.org/10.23641/asha.16583426

Many children are at risk for intelligibility deficits, including those with a wide range of neurodevelopmental disorders, genetic syndromes (e.g., Down syndrome, craniofacial syndromes), or hearing impairment (including children with cochlear implants or other assistive hearing devices). Even without other risk factors, about 10% of children have developmental speech sound disorders (Bishop, 2010), and many may experience reduced speech intelligibility as a result. Regardless of the cause, speech intelligibility deficits can have a negative impact on social participation, educational engagement and achievement, and quality of life (Dickinson et al., 2007; Fauconnier et al., 2009). Accurate and early identification of children with speech intelligibility that falls outside the range of age-based typical expectations is critical to ensure that children receive intervention to improve intelligibility.

Comprehensive, objective, empirically derived milestones for acquisition of intelligible speech are not, however, available at the present time, thus limiting our ability to identify children who fall outside the range of typical expectations for their age. Current standards for speech intelligibility development are based on parent report measures. Whereas parent report–based tools for characterizing acquisition of developmental milestones are widely used, a common criticism of such measures is a lack of normative data upon which milestones are based (Sheldrick et al., 2019). In the case of speech intelligibility, the most widely referenced standards for typical children were identified using categorical ratings by parents of how much of their child's speech they believed a stranger would be able to understand (Coplan & Gleason, 1988). Results indicated that, for children between 12 months and 5 years, the majority of parents felt that their children were (a) 50% intelligible by 22 months of age, (b) 75% intelligible by 37 months of age, and (c) nearly 100% intelligible by 47 months of age. However, these findings were not examined relative to empirically measured intelligibility data obtained directly from children. Therefore, the extent to which children objectively reach 50%, 75%, and 100% intelligibility by the ages of 22, 37, and 47 months is unknown. Similarly, McLeod et al. (2012, 2015) developed a seven-item parent report measure to characterize intelligibility in context for children. The Intelligibility in Context Scale (ICS; McLeod et al., 2015) is widely used clinically and has been translated to many languages. However, normative data for the ICS are limited, covering only a narrow age range (4–5.5 years). One notable finding from research on the ICS, however, is that parents tend to rate their child's intelligibility as highest for themselves and lowest for strangers. In their study examining children between 4 and 5.5 years, McLeod et al. (2015) found that the oldest children had significantly better ICS scores than younger children. However, effect sizes were very small, with age groups separated by one tenth of a point on a 5-point scale (i.e., mean ICS score for typical children between 48 and 54 months was 4.3; mean ICS score for typical children between 54 and 59 months was 4.4). Although the ICS has proven very useful as a descriptive index of intelligibility, age-based normative expectations for ICS scores across a full range of ages have not been established; thus, the ICS cannot be used to identify definitively whether a child's intelligibility is age appropriate. Furthermore, the ICS has not been examined relative to traditional measures of speech intelligibility involving objective quantification of listeners' ability to discern spoken words from the acoustic speech signal, a longstanding paradigm in the motor speech disorders literature for overcoming bias associated with subjective ratings. Consequently, there remain important questions concerning normative standards for speech intelligibility development.

In a recent study, we developed growth curves for speech intelligibility by age for single words and for multiword utterances in English-speaking children between 30 and 47 months based on objectively measured orthographic transcription results from unfamiliar listeners (Hustad, Mahr, Natzke, & Rathouz, 2020). We found that the average 36-month-old child had intelligibility that was only 50% for both single-word utterances and multiword utterances. There was considerable variability among children at the earliest ages, with the range of intelligibility scores spanning from 18% (5th percentile) to 74% (95th percentile) at 30 months of age for single words. We also found that single words were more intelligible than multiword utterances at the earliest ages, but that by 47 months (the oldest ages examined in that study), intelligibility of multiword utterances was significantly higher than that of single-word utterances. Other studies have indicated a clear intelligibility advantage for multiword utterances over single-word utterances (Hodge & Gotzke, 2014a; Hustad et al., 2012; Miller et al., 1951), but the developmental trajectory of this finding has not been determined.

In our study of 30- to 47-month-old typical children, we considered intelligibility of multiword utterances globally for each child based on unfamiliar listener orthographic transcription of utterances spoken by children. In that study, we developed and employed an imputation-weighting procedure to account for the fact that not all children were able to produce the target stimulus sentences for longer utterances. Given that the speech stimuli produced by children varied in length between two and seven words and given that the ability to produce longer utterances is a developmentally acquired skill, involving advancements in language, memory, and motor control, intelligibility performance on utterances of different lengths might be expected to vary. Previous studies of children with dysarthria have suggested that the intelligibility advantage for multiword utterances over single-word utterances may be impacted by sentence length. That is, the intelligibility benefit of multiword utterances seemed to plateau with four-word utterances, with no further benefit observed for longer utterances (Hustad et al., 2012). However, the earlier study only examined children at 4 years of age, and thus, the differential effects of utterance length on intelligibility by age and sentence length are not known.

A notable finding from our study of 30- to 47-month-old typical children (Hustad, Mahr, Natzke, & Rathouz, 2020) was that only the 95th percentile of children approached 100% intelligibility for multiword utterances by 47 months, and the average 47-month-old child was 78% intelligible. The vast majority of children had not yet plateaued in their development at 47 months. It is also notable, however, that the range of variability among children reduced with age. Comparable objective, empirically derived milestones for acquisition of intelligible speech beyond 47 months of age are currently not available, thus compromising our ability to identify older children who fall outside the range of typical expectations for their age.

Another area of interest for this study was the examination of growth rate and the age of maximum growth for children in different percentiles. In our work on speech intelligibility development in children with cerebral palsy with and without dysarthria, we found that children experienced maximum intelligibility growth between the ages of 36 and 60 months (Hustad, Sakash, Natzkie, et al., 2019). However, for children who had deficits in speech and language ability, results suggested that the age of maximum growth occurred in the later months of this time frame (Hustad, Mahr, Broman, & Rathouz, 2020). To our knowledge, analogous studies have not been performed on typically developing children, so we do not know about growth rates or the timing of peak growth in speech intelligibility. This information is critically important in order to provide a context for interpreting data from children with dysarthria and other developmental speech disorders and could serve as a foundation for considering critical periods of speech growth and the ways that such periods may be similar and different in children with delays and disorder conditions.

In this study, our first goal was to extend our earlier work by developing growth curves and developmental percentiles based on objective and direct measurement of speech intelligibility for typical children to the age of 119 months (9 years; 11 months). A primary concern was examination of average children (50th percentile) and of children on the lowest end of typical development (5th percentile) in order to establish minimal thresholds that could be used for identification of potential intelligibility impairment. We also present data showing the impact of utterance length on intelligibility by age, and we examine the global effect of utterance length (multiword vs. single words) on intelligibility over the course of development. Our second goal was to quantify growth rates and the ages at which children in different percentiles showed the most change in intelligibility. To do this, we employed data from our previous article (Hustad, Mahr, Natzke, & Rathouz, 2020) and added new data from an additional 374 typically developing children to develop growth curves for the full range of development using cross-sectional data from different children across a range of ages. A key outcome was to establish quantitatively derived benchmarks that can be used as age-specific reference guidelines for single-word and multiword intelligibility for speech-language pathologists and other health professionals to guide identification of children who fall outside the range of typical age-level expectations for intelligibility. Such information could ultimately be used to develop a tool for screening for intelligibility impairment or delay in children. Research objectives were as follows:

1. Estimate minimal and average expected thresholds in terms of percentiles of intelligibility development between the ages of 30 and 119 months in typical children separately for single-word and multiword productions.
2. Quantify differences in the age and rate of steepest intelligibility growth across the range of percentiles (i.e., compare the upper range of the intelligibility growth distribution to lower range of the distribution) to advance our understanding of how timing of growth differs.

Based on our work on younger typical children, which showed steady intelligibility growth to 47 months of age, we hypothesized that intelligibility would continue to grow through 119 months of age, but that variability among children would decrease with age, particularly as children approached the ceiling of intelligibility (100%). Also, based on our earlier work and based on studies of adults examining intelligibility of single words versus multiword utterances, we expected that there would be a consistent advantage for multiword utterances, but we did not have a hypothesis regarding the age at which this difference would become constant or stabilize. As part of this objective, we also report and visualize the effects of age and utterance length on intelligibility, although this analysis was more descriptive than confirmatory. Regarding the age of steepest growth, we expected that children would show the most rapid growth early in development, following our findings on children with cerebral palsy in previous studies. Also based on our studies of children with cerebral palsy, we expected typical children in the lowest percentiles to show their most rapid growth at slightly older ages than those in higher percentiles.

## Method

This study was reviewed and approved by the University of Wisconsin–Madison Institutional Review Board (Social and Behavioral Sciences). Informed consent was obtained on behalf of all participants. Methods reported in this article are identical to those employed in Hustad, Mahr, Natzke, and Rathouz (2020); therefore, abbreviated descriptions are provided here.

### Participants

#### Typically Developing Children

Typically developing children were recruited through public postings, including flyers posted in local venues and online advertisements, and from public schools in and around Madison, Wisconsin, via a research registry maintained by the Waisman Center Clinical Translational Core. Inclusion criteria were (a) American English as the primary language in the home, (b) hearing within normal limits as indicated by pure-tone hearing screening or distortion product otoacoustic emission screening bilaterally or by parent report for very young children who did not tolerate screening, (c) speech within normal limits as indicated by articulation scores on the Arizona Articulation Proficiency Scale–Third Edition (Fudala, 2001), and (d) language within normal limits as indicated by the Preschool Language Scales–Fifth Edition Screening Test (Zimmerman et al., 2012) or the Clinical Evaluation of Language Functions–Fifth Edition Screening Test (Wiig et al., 2013). Children receiving intervention services for any educational or developmental concern were excluded, as were those with any medical diagnoses related to development.

A community-based sample of 600 children between the ages of 30 and 119 months enrolled in this study (306 girls and 294 boys). Of these, 47 children failed one or more of the foregoing speech, language, and hearing screening inclusion measures and were therefore excluded from this study; 15 did not qualify due to either noncompliance during the data collection session or disclosure of information during the data collection visit related to native language status or developmental diagnosis that rendered the child not eligible for participation.

In total, speech samples from 538 typically developing children (282 girls and 256 boys) were included in this study. All children were from the upper midwestern region of the United States, and their demographic characteristics reflect those of Wisconsin. Table 1 shows demographic information for the children. Table 2 shows the age and sex distribution of children. We highlight that data from the youngest 164 children were published previously in our study examining intelligibility development between 30 and 47 months of age (Hustad, Mahr, Natzke, & Rathouz, 2020).

**Table 1.** Demographic characteristics of children (*N* = 538).

Characteristic	Male (*n = 257*)	Female (*n = 281*)
Race		
White	232 [10]	239 [6]
Black	5	2
Asian	3	6
American Indian	0	1
Native Hawaiian/Pacific Islander	0	1
More than 1 race	10 [1]	21 [2]
Other	0	0
Not reported	7	11
2-Factor Hollingshead Social Index mean	55.60 (7.98)	55.55 (7.93)
Maternal education		
Graduate degree or graduate professional training	119	139
Standard college or university degree	117	104
Partial college or specialized training	11	22
High school graduate	5	4
Not reported	5	12

*Note.* Number of additional children in this racial category whose parents identified them as having Hispanic ethnicity are indicated in []. All other children were identified as non-Hispanic. Standard deviations are indicated by ().

**Table 2.** Age and sex distribution of typically developing children.

Range (months)	Children *n*	Boys *n*	Girls *n*	*M*_age_ (months)
30–35	57	28	29	33
36–41	50	24	26	38
42–47	58	20	38	45
48–53	53	18	35	50
54–59	59	31	28	57
60–65	51	28	23	63
66–71	52	28	24	68
72–77	44	24	20	75
78–83	57	27	30	80
84–89	9	5	4	86
90–95	14	4	10	92
96–101	7	2	5	99
102–107	9	2	7	105
108–113	7	6	1	110
114–119	11	9	2	116

*Note.* Younger children were oversampled because of the variability demonstrated in our earlier study (Hustad, Mahr, Natzke, & Rathouz, 2020).

#### Adult Listeners

Normal-hearing naïve adult listeners were recruited through campus postings at the University of Wisconsin–Madison. Listeners orthographically transcribed the audio recordings of children in a sound-attenuating suite in our laboratory. Inclusion criteria were (a) hearing within normal limits as indicated by pure-tone hearing screening; (b) age between 18 and 45 years; (c) no more than incidental experience listening to or communicating with persons having communication disorders; (d) native speaker of American English; and (e) no identified language, learning, or cognitive disabilities per self-report. All listeners who enrolled completed the study. In total, 1,076 adults (280 men and 796 women) made intelligibility transcriptions of the children. Two different listeners heard each child (538 children × 2 listeners = 1,076 listeners); each listener heard only one child producing all stimulus material. The mean age of listeners was 20.8 years (*SD* = 3.7).

### Materials and Procedure

#### Acquisition of Speech Samples From Children

Children produced a standard set of speech stimuli, elicited by a research speech-language pathologist in a sound-attenuating suite at the Waisman Center. Speech stimuli were from the Test of Children's Speech Plus (TOCS+; Hodge & Daniels, 2007); all children produced the same corpus of words and sentences. See Supplemental Material S1 for the specific sentences and words employed in this study. TOCS+ stimuli were developed to be linguistically appropriate for children and have been used regularly in related research (Hodge & Gotzke, 2014a, 2014b; Hustad, Sakash, Broman, & Rathouz, 2019; Hustad, Sakash, Natzke, et al., 2019). Having children produce a known corpus of stimuli that was the same across all children allowed us to compare listener orthographic transcriptions against known target responses, thus ensuring that intelligibility scores were an accurate reflection of which target words were perceived correctly by listeners. Child productions were elicited for 38 single words and up to 60 sentences. Sentences systematically ranged in length from two to seven words, with 10 utterances of each length. Speech samples were obtained using elicitation procedures in which children repeated recordings of stimuli, while viewing an image depicting each word or sentence via an iPad. Note that not all children were able to produce utterances of each sentence length due to developmental constraints. The multiword protocol started with the 10 two-word utterances and advanced to the 10 three-word utterances and so on—stopping when the child was not able to produce all 10 utterances of the target length. Children's speech was recorded using a professional quality digital audio recorder (Marantz PMD 570) at a 44.1-kHz sampling rate (16-bit quantization) and a condenser studio microphone (Audio-Technica AT4040) positioned 18 in. from the child's mouth. Recordings were monitored in real time, and recording levels were adjusted on a mixer (Mackie 1202 VLZ) to obtain optimized recordings.

#### Acquisition of Intelligibility Data

Digital recordings of children's speech were prepared for playback to listeners, which involved separation of each utterance into its own file, removal of extraneous noises preceding or following production of each utterance, and peak amplitude normalization. All samples for each child were presented to two different listeners who transcribed orthographically what they heard in a self-paced task in a quiet room with peak audio output levels calibrated to 75 dB SPL. Child productions were presented in random order, with all single words presented in one block and all multiword utterances presented in a separate block; block order was counterbalanced. Listeners were permitted to hear each stimulus utterance only one time. Listeners were required to type orthographically what they thought the child said following each production.

Intelligibility scores were obtained by tallying the number of words transcribed correctly by each listener relative to the target words that children produced. As is usual in speech intelligibility research (Hodge & Gotzke, 2014a, 2014b; Yorkston & Beukelman, 1978, 1980), scores for each of the two independent listeners for each child were averaged. However, we considered single-word intelligibility separately from multiword intelligibility. Not all children were able to produce sentences of all lengths—in particular, some of the younger children were not able to produce the longest sentences. To correct for this problem and to ensure that intelligibility scores were not biased by this testing artifact, multiword intelligibility was calculated as a weighted average (number of words from two up to seven) of the proportion of words correctly transcribed for each utterance length using the imputation-weighting procedure described in Hustad, Mahr, Natzke, and Rathouz (2020). Briefly summarized, in this procedure, we trained regression models to predict an intelligibility for utterance length *L* using a child's longest utterance length and the intelligibility for each of the shorter utterance lengths *l* < *L*, and the predictions from these models were used to impute intelligibility for missing utterance lengths. For example, a child who only reached five-word utterances would have their six-word intelligibility predicted from the one-, two-, three-, four-, and five-word intelligibility and the length of the longest utterance (five). This imputed value would be carried forward to impute seven-word intelligibility. After imputation, each child has one intelligibility score for each utterance length, and we compute a child's multiword intelligibility as a weighted average of their two- to seven-word intelligibilities, where the weights are based on the probability of producing that utterance length at that child's age. For the youngest children, six- to seven-word utterance are down-weighted to practically zero because children did not produce utterances of this length, but for older children, all lengths are equally weighted.

We calculated the interrater reliability of intelligibility measurements by examining the agreement between percentages of words correctly transcribed for each listener of each child. To do this, we used the intraclass correlation coefficient (ICC), estimated using the irr R package (Version 0.84.1; Gamer et al., 2019). Each of the 538 participants were transcribed by two different listeners (1,076 unique listeners). We used an average score, agreement-based, one-way random effects model (Shrout & Fleiss, 1979), and we found strong agreement among listeners for average multiword intelligibility scores, ICC = .98, 95% confidence interval (CI) [.98, .99], and among listeners for average single-word intelligibility scores, ICC = .95, 95% CI [.94, .96].

### Data Analysis

Single-word and multiword intelligibility were analyzed separately using the same modeling approach. We used a beta regression model to estimate the mean and variability of intelligibility as a function of age. The beta distribution is appropriate for continuous data ranging from a minimum (0%) to a maximum (100%) especially in the presence of strong floor or ceiling effects. The beta distribution used in this analysis is governed by two parameters—location (mean intelligibility) and precision (related to the number of trials or the denominator in the modeled proportions). We modeled each of these parameters as a function of age, using a 3-*df* natural cubic spline on the logit scale for the mean parameter and a second 2-*df* natural cubic spline on the logit scale for the scale parameter. These natural cubic splines allowed the mean and scale to vary flexibly with age.

**Figure 1.**
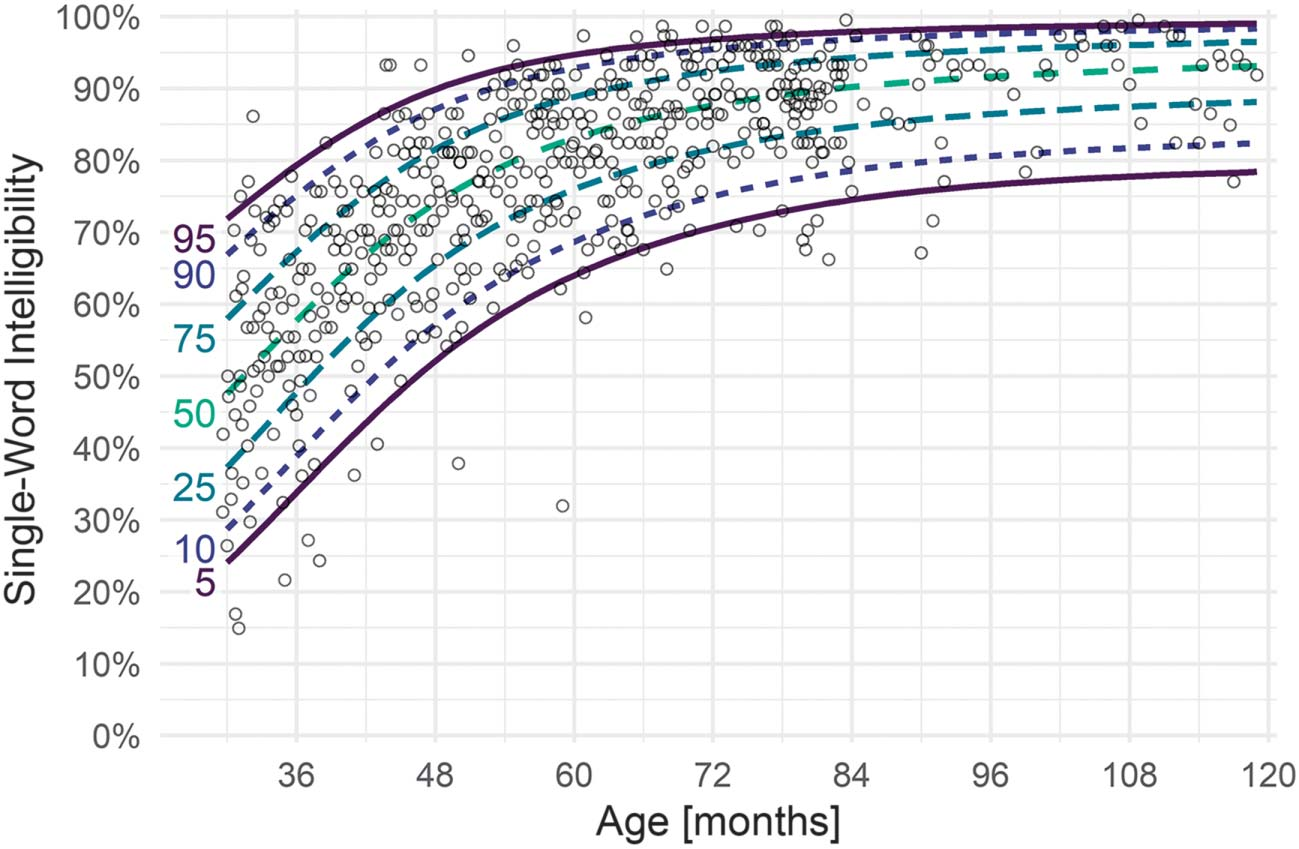
Quantile growth curves based on location-scale beta regression models for single-word intelligibility. *N* = 538 typically developing children.

**Figure 2.**
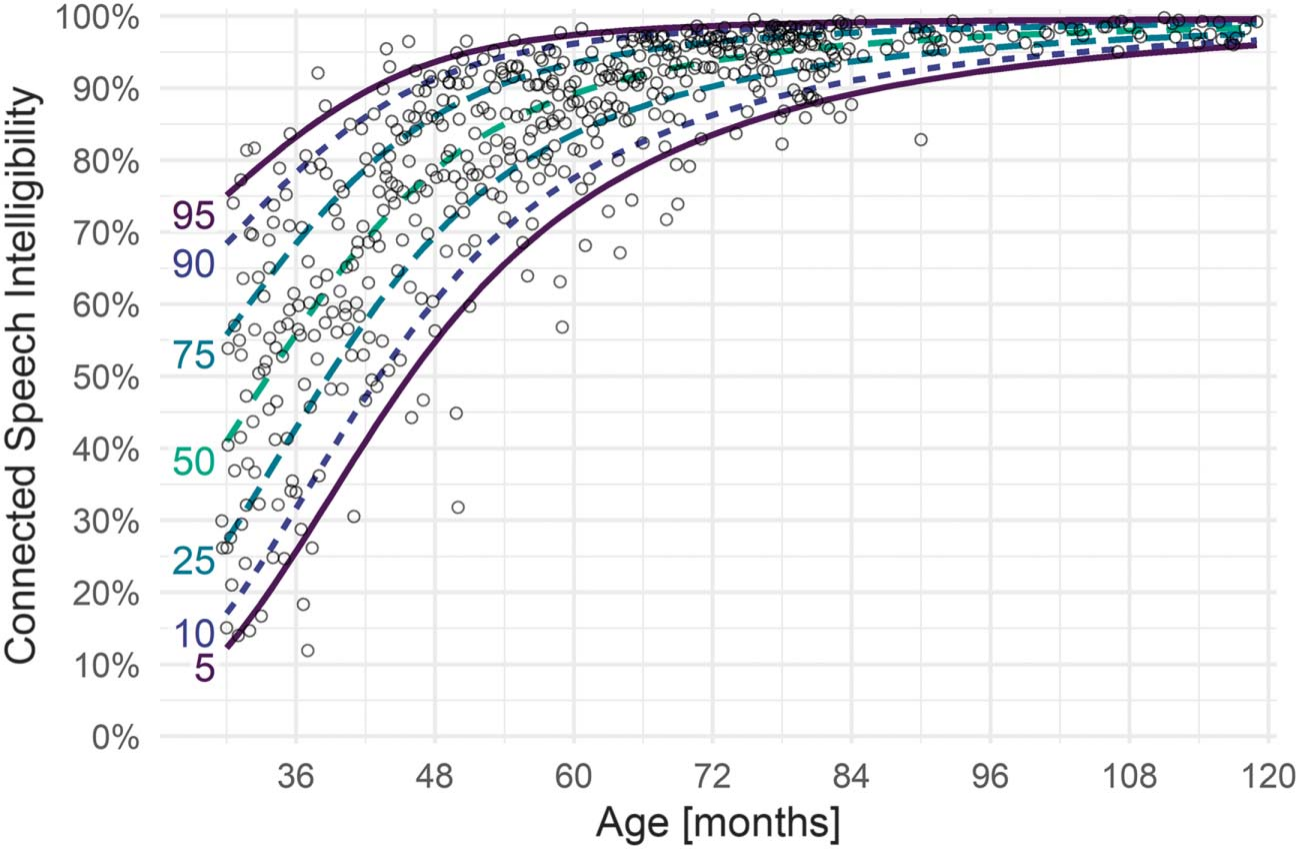
Quantile growth curves based on location-scale beta regression models for multiword intelligibility. *N* = 538 typically developing children.

After confirming via model diagnostics that the beta yielded an appropriate fit to the data, we used the estimated location precision parameters to extract estimated quantiles of intelligibility by age and thereby to compute quantile growth curves (see Figures 1 and 2). For example, at 36 months, our model for single-word intelligibility estimated a mean of .57 (57% intelligibility), with a precision of .3. Given these parameter estimates, we use the beta quantile function to estimate intelligibility at the 5th (34%), 10th (39%), and other percentiles. We computed the age at—and rate of—steepest growth at each percentile by computing the derivative of the growth curve and numerically finding the maximum.

In order to estimate the uncertainty in the estimated growth curves, we computed 95% CIs for estimates by sampling from the multivariate normal distribution associated with the estimate model parameters and the corresponding robust variance–covariance matrix. We sampled 10,000 new parameter values from a multivariate normal distribution, and for each draw, we recomputed the quantile growth curves. For the previous example of single-word intelligibility at 36 months, we therefore had 10,000 estimates of the 5th percentile (and other percentiles), and we computed CIs by taking the .025 quantile (30%) and the .975 quantile (38%) of these estimates.

Our primary research objective was the estimation of developmental growth curves for speech intelligibility. We also performed follow-up analyses to report and visualize the simple effects of age and utterance length on intelligibility. These analyses used only the observed data without imputation or weighting, and the number of observations varied by age and utterance length cells. Given the uneven data and a potential selection effect (where younger children who reached longer utterance have higher overall intelligibilities), our analysis here was descriptive. Analyses were carried out in R (Version 4.0.3; R Core Team, 2019) using the gamlss package (Version 5.2.0; Rigby & Stasinopoulos, 2005).

## Results

Objective 1: Estimate minimal and average expected thresholds in terms of percentiles of intelligibility development between the ages of 30 and 119 months in typical children separately for single-word and multiword productions.

Figures 1 and 2 show the model-estimated growth curves for single-word and multiword utterances for 5th, 10th, 50th, 90th, and 95th percentiles of children. Table 3 summarizes modeled results by percentiles at half-year intervals; Table 4 summarizes observed means and standard deviations at half-year intervals. Descriptively, results indicate considerable variability among children of the same age across the various percentile curves until about 60 months of age, when the range begins to narrow. Variability in single-word intelligibility is greater than for multiword intelligibility throughout the age span. For single-word intelligibility, variability is relatively stable after 84 months of age (7 years), with the range between the lowest (5th) and highest (95th) percentiles maintaining about a 20-percentage-point difference. For multiword intelligibility, children also become more homogeneous after 84 months (7 years), with negligible differences between the lowest and highest percentiles by 102 months of age (8 years 6 months).

**Table 3.** Model-estimated intelligibility scores by percentile and age for single-word and multiword utterances (*N* = 538 typically developing children).

Age (months)	Single-word percentiles					Multiword percentiles				
	5th	10th	50th	90th	95th	5th	10th	50th	90th	95th
30–35	24.0	28.7	47.5	66.9	71.8	12.3	17.0	40.9	68.4	75.1
36–41	33.8	38.8	57.7	75.1	79.3	25.7	31.7	55.9	78.2	83.2
42–47	43.5	48.7	66.8	82.0	85.4	40.9	47.1	68.9	86.0	89.5
48–53	52.1	57.2	74.0	87.1	89.9	54.7	60.4	78.6	91.2	93.6
54–59	58.9	63.8	79.3	90.5	92.8	65.5	70.4	85.0	94.4	96.0
60–65	64.0	68.7	83.1	92.8	94.7	73.5	77.5	89.2	96.2	97.3
66–71	67.8	72.3	85.7	94.4	95.9	79.2	82.6	91.9	97.3	98.1
72–77	70.7	75.0	87.7	95.5	96.8	83.5	86.2	93.7	97.9	98.6
78–83	72.9	77.0	89.1	96.2	97.4	86.7	89.0	95.0	98.4	98.9
84–89	74.5	78.6	90.2	96.8	97.9	89.1	91.0	96.0	98.7	99.1
90–95	75.7	79.7	91.0	97.2	98.2	91.0	92.6	96.7	98.9	99.3
96–101	76.6	80.6	91.6	97.6	98.4	92.5	93.8	97.2	99.1	99.4
102–107	77.3	81.2	92.1	97.8	98.6	93.7	94.7	97.6	99.2	99.4
108–113	77.7	81.7	92.5	98.0	98.8	94.6	95.5	97.9	99.3	99.5
114–119	78.1	82.1	92.8	98.2	98.9	95.4	96.2	98.2	99.3	99.5

*Note.* Values within each cell are expressed in % intelligibility.

**Table 4.** Mean observed intelligibility scores and standard deviations by age for single-word and multiword intelligibility.

Age (months)	Children *n*	Single-word intelligibility (%)		Multiword intelligibility	
		*M*	*SD*	*M*	*SD*
30–35	57	51.5	15.8	48.8	19.6
36–47	108	63.5	13.0	67.4	17.0
48–59	112	73.7	10.9	83.2	10.6
60–71	103	79.8	8.8	89.8	6.9
72–83	101	83.6	7.2	94.5	3.8
84–95	23	83.3	8.6	96.0	3.7
96–119	34	86.6	5.9	97.7	1.3

*Note.* We collapse across older age bands because of sparser sampling in those bands.

Table 5 shows the age at which children are expected to achieve 50%, 75%, and 90% intelligibility thresholds for average (50th percentile) and lower performing (5th percentile) children based on modeled results. For children in the 50th percentile, we expect single-word intelligibility to have reached 50%, 75%, and 90% thresholds by 31, 49, and 83 months, respectively. We expect multiword intelligibility to have reached 50%, 75%, and 90% thresholds by 34, 46, and 62 months, respectively.

**Table 5.** Model-estimated age (months) of achieving 50%, 75%, and 90% intelligibility thresholds for 50th (average) and 5th (lower) percentiles of typically developing children for single-word productions and multiword productions.

Word production	Intelligibility	50th percentile (95% CI)	5th percentile (95% CI)
Single words	50%	31 [≤ 30, 33]	46 [44, 49]
	75%	49 [47, 51]	87 [78, ≥ 120]
	90%	83 [75, 91]	—^a^
Multiword utterances	50%	34 [32, 35]	46 [44, 47]
	75%	46 [44, 47]	61 [59, 64]
	90%	62 [60, 63]	87 [83, 91]

*Note.* 95% Confidence intervals for age are provided in brackets.

a Estimated age is ≥ 120 months.

For children in the 5th percentile, we expect single-word intelligibility to have reached 50%, 75%, and 90% thresholds by 46, 87, and 120 months, respectively. We expect multiword intelligibility to have reached 50%, 75%, and 90% thresholds by 46, 61, and 87 months, respectively.

As would be expected, our previous analysis of children between 30 and 47 months (*n* = 164; Hustad, Mahr, Natzke, & Rathouz, 2020) who are also included in this study produced highly similar results. However, in that work, the estimated 5th percentile curve resulted in 6.7% of the data falling below that curve. In the present analysis, that number is 4.3%. We have investigated this phenomenon and isolated the minor statistical modeling discrepancy to our use in the earlier article of the normal (Gaussian) response distribution versus the beta response distribution in the present analysis. Owing to the ceiling effect at 100% intelligibility, which is more salient for children at older ages, the beta model provides an improved fit to the data relative to the Gaussian model in the earlier article, when the larger range of ages is considered.

The quantile growth curves presented in Figures 1, 2, and 5 used a weighted average of intelligibility across utterance lengths. As described above, we imputed missing observations for utterance lengths where a child could not produce those utterances, and we weighted each of the utterance lengths based on the probability of reaching that utterance length at a given age. For completeness, we also examined simple age and utterance lengths on the observed, unweighted data. Observed intelligibility scores for age groups are presented in Table 3 (averaging over utterance lengths for multiword intelligibility).

**Figure 3.**
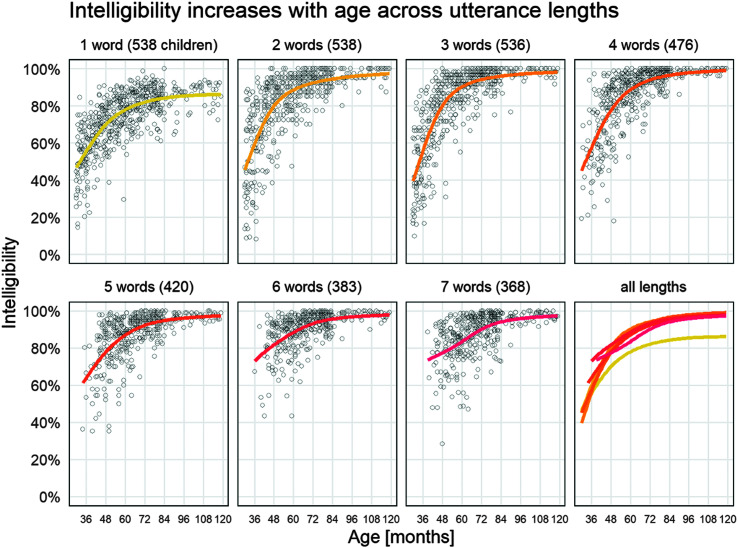
Intelligibility by age for each utterance length. Each point in each panel represents one child's average intelligibility for utterances of that length. The smooth line is the mean intelligibility estimated using a flexible beta regression model as used in the quantile growth curves. A separate model was estimated for each panel. Only observed data points are included, so the number of points starts at 538 children for single words and ends at 368 children with seven-word utterances.

Figure 3 shows the beta regression growth curve estimated separately for each utterance length. In all lengths, the observations followed a funnel pattern with a wider range of intelligibility at younger ages and a narrower range at older ages. Moreover, for all utterance lengths, mean intelligibility increased with age but showed decelerating trajectories (i.e., the curves plateaued). There was a clear difference between for single-word and multiword intelligibility; we discuss this difference in greater detail below.

**Figure 4.**
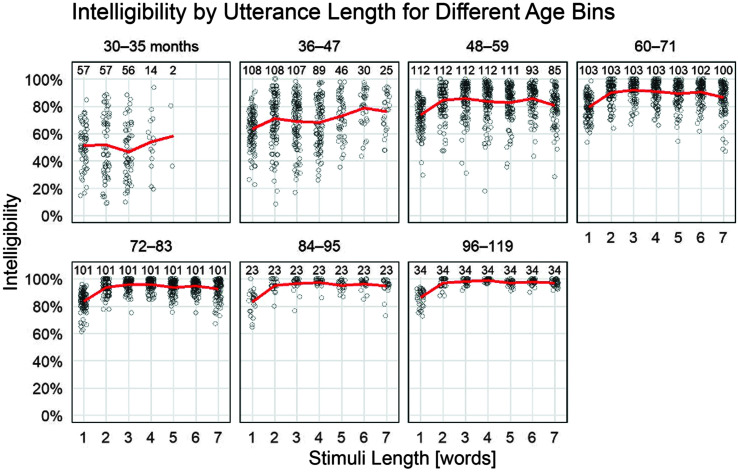
Effects of utterance length on intelligibility. The plot shows one point per child per utterance length; this point is the child's average intelligibility for items of that length. Only observed data points are included. The numbers above the points report the number of children in that column of points. For example, 108 children 36–47 months in age produced single words, but only 25 reached the seven-word utterances on the task. Lines connect the mean intelligibilities in each column.

Figure 4 shows intelligibility for different age groups as a function of utterance length (top) and as a function of a child's longest utterance length (bottom). For the panels containing children ages 48 months and older, there was a consistent change in the average intelligibility from one-word utterances to two-word utterances, but afterward, there was not a clear difference among the multiword utterance lengths. Of note, however, is that, in Figure 3, the starting intelligibility levels for the five-, six-, and seven-word growth curves appear to have shifted upward, such that longer utterance lengths have higher starting intelligibilities. This apparent age-by-length effect was, in fact, a selection effect in the task: Children who could reliably repeat longer utterances at earlier ages tended to have higher intelligibility than children who could not.

**Figure 5.**
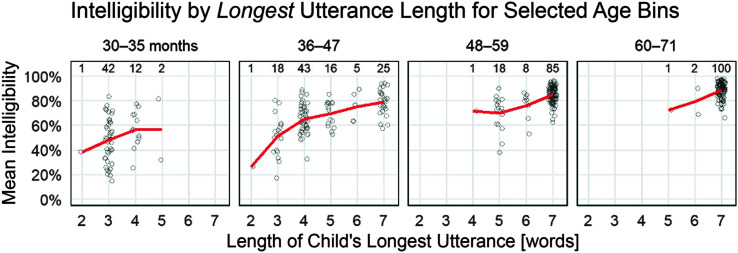
Task ceilings and intelligibility. Children are grouped by the length of their longest utterance. For example, in the 30–35 months panel, 42 children had a length of longest utterance of three words; they could not reliably repeat four-word utterances or longer. The plot shows one point per child; this point is the child's average intelligibility across all utterances. Only observed data points are included. Lines connect the mean intelligibilities in each column.

Figure 5 highlights this effect by illustrating how children who produced longer utterances within the elicited production task had higher intelligibilities on average. For example, among the 36- to 47-month-olds, the 25 children who reached seven-word utterances had a higher average intelligibility (*M* = 79%, *SE* = 2.1) than the 43 children who only reached four-word utterances (*M* = 65%, *SE* = 1.8). Once children could reliably repeat all of the utterances across all lengths, at approximately 60 months and older, utterance length did not have a clear effect on intelligibility.

**Figure 6.**
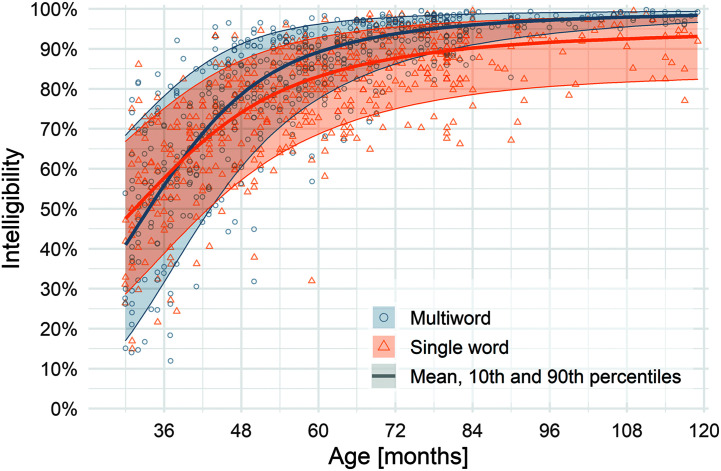
Growth trajectories of single words versus connected speech. Shaded regions reflect the range between the 10th and 90th percentiles.

Intelligibility of multiword utterances versus single-word utterances is shown in Figure 6. Multiword utterances had higher intelligibility than single-word utterances from 48 months through 119 months for all percentiles. For children in the 50th percentile, this difference was constant at about a 5% advantage for multiword utterances through 119 months of age. To test the constancy of the intelligibility difference after 48 months, we regressed the multiword versus single-word intelligibility difference onto age in months for just the participants who were 48 months and older (*n* = 373). The estimated month-by-month change in age was 0.02 percentage points, 95% CI [−0.03, 0.06]. This CI supports either positive or negative slopes with a magnitude of less than 0.1 percentage points per month, so we conclude that there was no long-term change in the average difference between multiword and single-word intelligibility. The difference between single-word and multiword intelligibility was considerably larger for children in the lower percentiles than those in the higher percentiles, particularly after about 60 months of age.

Objective 2: Quantify differences in the age and rate of steepest intelligibility growth across the range of percentiles (i.e., compare the upper range of the intelligibility growth distribution to lower range of the distribution) to advance our understanding of how timing of growth differs.

For children in the 50th percentile, the age of steepest growth was 33 months for both single-word and multiword intelligibility, and 95% CIs were [30, 32] for single words and [30, 33] for multiword utterances. These intervals are left-truncated because the lower bound age of children in the sample was 30 months. The rate of maximum growth was 1.7 [1.3, 2.0] intelligibility percentage points per month for single words and 2.5 [1.3, 2.0] intelligibility percentage points per month for multiword utterances.

For children in the 5th percentile, the age of steepest growth for single words was 36 [35, 38] months, with a rate of change of 1.7 [1.3, 2.0] intelligibility percentage points per month. For multiword utterances, the age of steepest growth was 39 [38, 41] months, with a rate of 2.5 [2.2, 2.9] intelligibility percentage points per month. We emphasize here that the term *growth* in all instances refers to cross-sectional differences across ages for children falling within the same percentile.

## Discussion

In this study, we provide the first systematic account of speech intelligibility development for single words and connected speech in typical children across the time frame from 30 to 119 months of age, creating novel growth curves quantifying typical developmental expectations. We examined speech intelligibility development based on directly measured objective speech data from 538 typically developing children using a cross-sectional research design. Our primary concern was examination of average children (50th percentile) and of children on the lowest end of typical development (5th percentile) in order to establish minimal thresholds that could be used for identification of potential intelligibility impairment. Therefore, our discussion is focused on results for quantiles in the lower half of typical development. We emphasize that our findings reflect intelligibility in controlled production and listening environments, which may yield different data than would be observed in real-life interaction. With that caveat, key findings of this research are that when objective measures of intelligibility are obtained from unfamiliar listeners, children reach quantitatively derived intelligibility thresholds of 50%, 75%, and 90% more than a year later than previous work based on subjective parent impressions (Coplan & Gleason, 1988) has suggested. In addition, intelligibility is still developing well into middle childhood, although there is considerable variability among children of the same age, especially in the younger years. Results also showed that there was a constant advantage for multiword intelligibility over single-word intelligibility from about 4 years on and that utterances of any length above one word evidenced a similar advantage. Finally, the maximal rate of change for intelligibility growth is consistent among children, but the age of steepest growth occurs later for children in lower percentiles. These findings are discussed below.

### Age of Reaching Intelligibility Thresholds and Impact of Utterance Length on Intelligibility

Previous research examining intelligibility development in typical children has been very limited and has primarily used subjective methods to characterize intelligibility, identifying benchmarks using indirect impressionist indices via parent ratings of children's performance (see Coplan & Gleason, 1988). That early work suggested that children should be nearly 100% intelligible by 4 years of age, 75% intelligible by 3 years of age, and 50% intelligible just before 2 years of age. In contrast, directly measured objective findings for multiword intelligibility from this study, in which unfamiliar listeners orthographically transcribe children's speech, indicate that average (50th percentile) children do not approach these thresholds until a year later. Thus, we do not expect average children speaking in multiword utterances to be above 90% intelligible until about 5 years of age, 75% intelligible until about 4 years of age, and 50% intelligible until about 3 years of age. Similarly, children in the 5th percentile are expected to reach these same thresholds about 1–2 years later than their average peers: 90% intelligibility at about 7 years of age, 75% intelligibility at about 5 years of age, and 50% intelligible at about 4 years of age.

By definition, children in the lower percentiles are below average (median) in their speech intelligibility. However, this difference is large for single-word intelligibility and narrower but nonnegligible for multiword utterances. Results of this study clearly highlight that there is a wide range of typical performance for both single-word intelligibility and multiword intelligibility until about 5 years of age, when children begin to become more homogeneous. Our previous work highlighted this broad range of variability to 4 years of age and demonstrated that intelligibility was higher for multiword utterances than for single-word utterances after 40 months of age (Hustad, Mahr, Natzke, & Rathouz, 2020). In this study, we extend this finding through 9 years, demonstrating that single-word intelligibility is consistently lower than multiword intelligibility by about 5% beginning around 4 years and continuing through 10 years of age based on unfamiliar listener orthographic transcriptions of children's speech. Results from this study further refine our understanding of the impact of utterance length on intelligibility through the observation that, after 4 years of age, intelligibility is not differentially affected by utterance length for children of the same age. That is, two-word utterances have the same advantage over single-word utterances as those observed for four-, five-, or six-word utterances.

Our descriptive analysis on intelligibility in different utterance lengths adds some more information to the developmental story. First, the same general developmental trajectory was observed within each utterance length: Children start highly variable but eventually narrow and plateau after 6 years of age. Simple visualization of intelligibility by age suggests a beneficial effect of utterance length where five-, six-, and seven-word utterances are more intelligible on average at 36 months of age than two-, three-, and four-word utterances. However, this length effect was a selection effect: Children who could reliably produce longer utterances at young ages tended to have higher overall intelligibility, which was consistent across all utterance lengths. On the other hand, children with lower intelligibility were less likely to be able to complete the elicited production tasks for longer utterances. Thus, it was not necessarily the case that longer utterances were more intelligible for young children. That said, there was a robust advantage for multiword utterances over single words starting at 48 months.

A key explanation for this difference between single-word and multiword intelligibility is the role of linguistic-contextual information provided by sentences. That is, context allows listeners to infer missing information, compensating for immature production features when making sense of children's speech. When linguistic context is reduced, in the case of single-word productions, listeners must rely to a greater extent on the speech signal itself, which in turn requires production features of the signal to be closer to an adult standard in order to be correctly deciphered into the intended word units. In the English language, there is variability in research findings regarding age of acquisition and mastery for different consonants and consonant clusters. However, a recent comprehensive review article suggests that most consonants are acquired by about 5 years of age, but mastery of all speech sounds is not expected until about 7 years of age (Crowe & McLeod, 2020). This article is the first to demonstrate that, on average, children are expected to be 90% intelligible for single words at about the same time that speech sounds are mastered, but that the 90% intelligibility threshold is reached several years earlier (around 5 years) for multiword utterances, before children have mastered all speech sounds, but consistent with the timing of mastery for the majority of consonants (Crowe & McLeod, 2020). This finding highlights the relationship between functional abilities (characterized by intelligibility) and underlying skill development (characterized by acquisition of adultlike speech sounds).

### Rate of Growth

A second key finding from this study is that intelligibility peaks in its rate of growth at very early ages and that children in lower developmental percentiles tend to be older when they experience their peak in growth rate. The finding that children with lower levels of speech performance experience peak growth later than children with higher levels of performance is generally consistent with findings from our research on children with cerebral palsy (Hustad, Mahr, Broman, & Rathouz, 2020). Interestingly, findings from this study indicate that the 5th percentile of typical children demonstrate peak growth at the same time as children with cerebral palsy who have no evidence of speech motor involvement, providing an important point of convergence for these two groups of children.

In this study, we also found that the rate of growth between ages based on our cross-sectional data was the same for children in average and lower percentiles. We note, however, that children in lower percentiles simply experienced their growth at later ages. Thus, the main differences among typical children in different intelligibility by age percentiles are the starting point for intelligibility at 30 months and the timing of steepest growth, which together result in later ages for crossing key intelligibility thresholds of 50%, 75%, and 90%.

Findings from this study regarding age of steepest intelligibility growth and rate of growth for single words and multiword utterances provide a foundation for beginning to consider timing of intervention for children with speech intelligibility deficits. For example, given some sense of when children can be expected to be growing the most quickly in terms of speech intelligibility, an important next step is to determine how children with different disorders may be similar and different in terms of growth parameters. Our work on children with cerebral palsy clearly indicates that children with more extensive speech and language challenges have their peak growth at much later ages than typical children (Hustad, Mahr, Broman, & Rathouz, 2020). We do not know whether this same finding holds true for other populations of children with speech disorders. Furthermore, the question of whether intervention provided during windows of accelerated growth might yield an extra boost to development or whether intervention is best delivered prior to or following periods of accelerated growth is unknown, but there are likely important clinical implications for this information.

### Limitations and Future Directions

This study used a cross-sectional methodology to develop growth curves; therefore, we could not examine individual child-level growth trajectories. Studies that employ longitudinal designs could reveal important information about individual patterns of growth that refine our ability to explain variability among children.

Only typically developing children were included in this study. Validation of cut-points requires analyses that include children with known atypical development. Future work will begin to examine receiver operating characteristic curves that allow characterization of sensitivity and specificity of intelligibility scores for differentiating between groups of children with typical and atypical development.

Our intelligibility measurement is a laboratory ideal where context was minimized so that the speech signal had to convey all of the meaning for each word or utterance. Therefore, the percentiles identified here set a lower bound on a child's functional intelligibility, because any additional context (familiarity with the speaker, knowledge about the communication topic, and extralinguistic cues) would likely serve to boost intelligibility. In everyday speech, a child might be more intelligible than our percentiles indicate.

The study of speech intelligibility development informs us about functional speech capability and how it develops, but it does not specify the precise underlying variables that are responsible for change with age. Articulation development and the integrity of individual speech sounds are clearly critical to intelligibility, but the contributions of individual phonemes and the precision of their production to intelligibility are unknown. This knowledge could shift the way that we think about intervention, allowing us to adjust treatment target priorities based on which phonemes contribute most to intelligibility deficits. Research of this nature is necessary to advance the study of speech development and to identify the specific underlying sources of intelligibility changes.

This study examined children in the upper midwestern region of the United States. Sampling reflected demographics of the region, which comprised many middle-class families who were well educated. Findings may be different with a more diverse sample. Nevertheless, our findings from a large-scale community-based sample provide novel information that serves as a foundation for informing clinical practice related to intelligibility development in children.

### Clinical Implications

Development of intelligibility is highly variable even across typically developing children of the same age; however, at their peak growth, children tend to grow at the same rate, regardless of where they fall on the developmental growth distribution. Variability decreases as children get older, especially for multiword utterances. General rules of thumb for intelligibility of multiword utterances, based on data for the lowest performing typical children, are as follows: (a) By 4 years, children should be at least 50% intelligible to an unfamiliar listener; (b) by the 5 years, children should be at least 75% intelligible to an unfamiliar listener; and (c) by just over 7 years, children should be 90% intelligible to an unfamiliar listener. These milestones result from standardized research measurement, and clinical tools for obtaining parallel measures are not currently available. However, mobile app–based automated evaluation of speech intelligibility is on the horizon, and a key goal is to make speech intelligibility measurement readily accessible for screening and other clinical environments.

## Supplementary Material

10.1044/2021_JSLHR-21-00142SMS1 Supplemental Material S1 Stimuli selected from the Items in the 2-, 3-, 4-, 5-, 6- and 7-Word Length Pools of the TOCS+ Sentence Intelligibility Measure.
