# Supplementary material for: Speech Development Between 30 and 119 Months in Typical Children I: Intelligibility Growth Curves for Single-Word and Multiword Productions
Source: J Speech Lang Hear Res. 2021 Sep 7;64(10):3707–19. doi: 10.1044/2021_JSLHR-21-00142 (PMC9132140; doi:10.1044/2021_JSLHR-21-00142)
Supplement: Supplemental Material S1 [file JSLHR-64-3707-s001.pdf]

Supplemental Material S1. Stimuli selected from the Items in the 2-, 3-, 4-, 5-, 6- and 7-Word Length Pools of the TOCS+ Sentence Intelligibility Measure.[a]

2-word utterances

1. Cowboy boots
2. Bird house
3. Say goodbye
4. Big potato
5. Baby sock
6. Doctor bag
7. Get off
8. Hug daddy
9. Living room
10. Animal crackers

3-word utterances

1. Gather the toys
2. Five more cookies
3. Tie those shoes
4. Make a birdhouse
5. Cook big hotdogs
6. Point to Teddy
7. Take his turn
8. Open the house
9. That's not white
10. Put these together

4-word utterances

1. Wear a cowboy hat
2. She could hide here
3. That is her dog
4. He likes potato chips
5. Get them some coffee
6. Cut two small pieces
7. Both faces are happy
8. Find the brown lid
9. She chewed her fingers
10. Jump over the box

5-word utterances

1. The sign says 'keep out'
2. Tie up the garbage bag
3. Baby likes his new toy
4. They'll eat those hotdogs soon
5. Water shoots from that gun
6. This cheese doesn't smell good
7. They are singing 'Happy Birthday'
8. Give the flowers some water
9. His fingers are in wrong
10. Put all the toys away

6-word utterances

1. The cowboy hat has a feather
2. Make a check beside the '2'
3. Taste the cookies that she baked
4. Take the lid off the pot
5. She laughed at his funny jokes
6. That baby is learning to talk
7. He gave Mommy a birthday card
8. He wants somebody to push him
9. He winds up the toy ghost
10. There's a playground near school

7-word utterances

1. She got mad and pushed the boy
2. She is showing him how to jump
3. The bird landed beside the shaggy dog
4. Pour some tea for both of us
5. Baby reached to get another chocolate cookie
6. Fill the fridge with things to eat
7. Be very quiet when Baby is sleeping
8. The loud noise scared the cat away
9. They ate birthday cake and drank pop
10. Don't let go or it will fall

Stimuli Selected from the Pool of Practice Words and Form 1 of the *TOCS+ Word Intelligibility Measure*

1. Pizza
2. Hug
3. Boot
4. Ball
5. Bee
6. Buddy
7. Hoot
8. Mud
9. Snow
10. Chew
11. Pan
12. Come
13. Boy
14. Bad
15. Hot
16. Gum
17. Beanie
18. Zoo
19. Hat
20. Seat
21. Yawn
22. No
23. Full
24. Bus
25. Jar
26. Top
27. Lock
28. Walk
29. Bow
30. Eat
31. Sheet
32. rock

To learn about the TOCS+ software, please see:

Hodge, M. M., & Gotzke, C. (2014). Construct-related validity of the TOCS+ measures: Comparison of intelligibility and speaking rate scores in children with and without speech disorders. *Journal of Communication Disorders*, *51,* 51–63. https://doi.org/10.1016/j.jcomdis.2014.06.007

[a]Reprinted with permission.
